# Supplementary material for: Identifiability of phenotypic adaptation from low-cell-count experiments and a stochastic model
Source: PLoS Comput Biol. 2025 Jun 24;21(6):e1013202. doi: 10.1371/journal.pcbi.1013202 (PMC12204626; doi:10.1371/journal.pcbi.1013202)
Supplement: S1 File — (PDF) [file pcbi.1013202.s001.pdf]

S1 IBM/CME comparison

In Fig. A we compare the probability mass functions arising from the solution of the CME to the empirical distribution arising from 1000 cell proliferation assays simulated using the IBM.

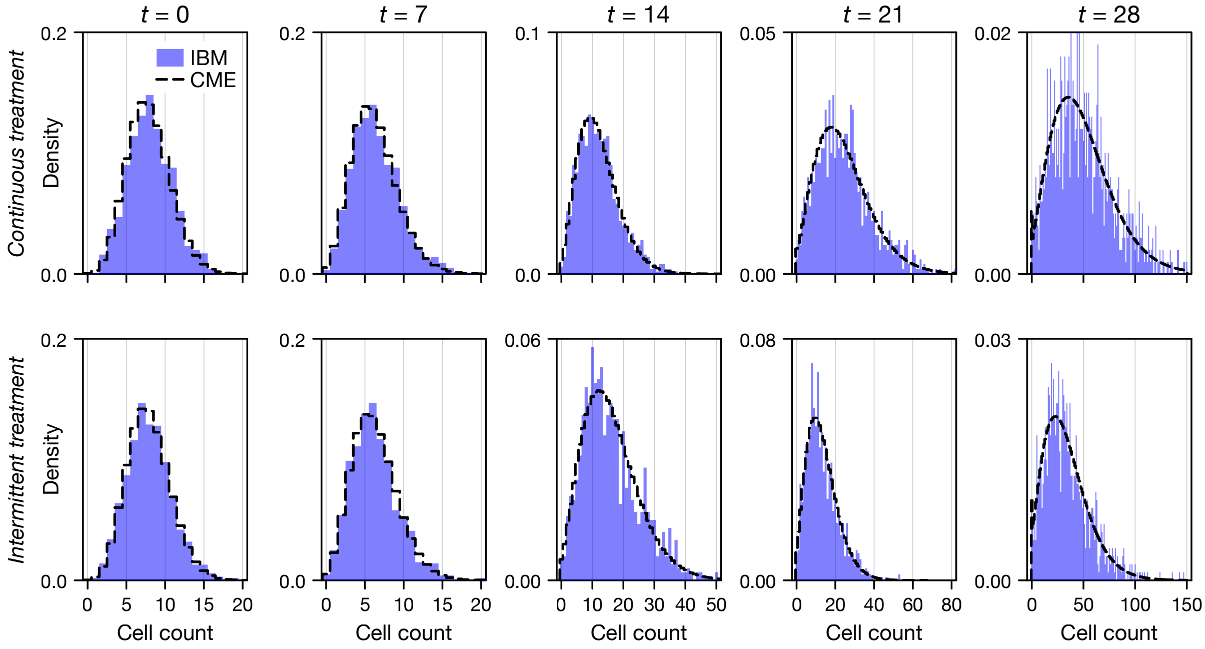

Figure A. Chemical master equation comparison. Comparison between $n = 1000$ realisations of the IBM (blue) and the solution to the CME (black dashed) under continuous treatment (top row) and intermittent treatment (bottom row). All parameters are consistent with those in Fig. 2 of the main text.
