# Supplementary material for: Identifiability of phenotypic adaptation from low-cell-count experiments and a stochastic model
Source: PLoS Comput Biol. 2025 Jun 24;21(6):e1013202. doi: 10.1371/journal.pcbi.1013202 (PMC12204626; doi:10.1371/journal.pcbi.1013202)
Supplement: S2 File — (PDF) [file pcbi.1013202.s002.pdf]

S2 MCMC priors and results

The priors for each parameter are given in Table A. In Fig. A, we show marginal posterior distributions for all parameters corresponding to the analysis in Fig. 3 of the main text. In the main text, we present results for experiment termination times $t = \{1\,\mathrm{d}, 3\,\mathrm{d}, 5\,\mathrm{d}, 7\,\mathrm{d}\}$. In Fig. A, we additionally consider various other termination time sets, as indicated.

Table A. Parameters, parameter descriptions, and parameter priors used for analysis in the main text.

Parameter	Description	Prior
γ_1	Sensitive ($x = 0$) growth rate off drug	Uniform$(-1, 1)$
γ_2	Sensitive ($x = 0$) growth rate on drug	Uniform$(-1, 1)$
γ_3	Resistant ($x = 1$) growth rate off drug	Uniform$(-1, 1)$
γ_4	Resistant ($x = 1$) growth rate on drug	Uniform$(-1, 1)$
$\log \nu$	Adaptation speed	Uniform$(-6, 1)$
$\log \beta$	Heterogeneity/diffusivity parameter	Uniform$(-6, -1)$

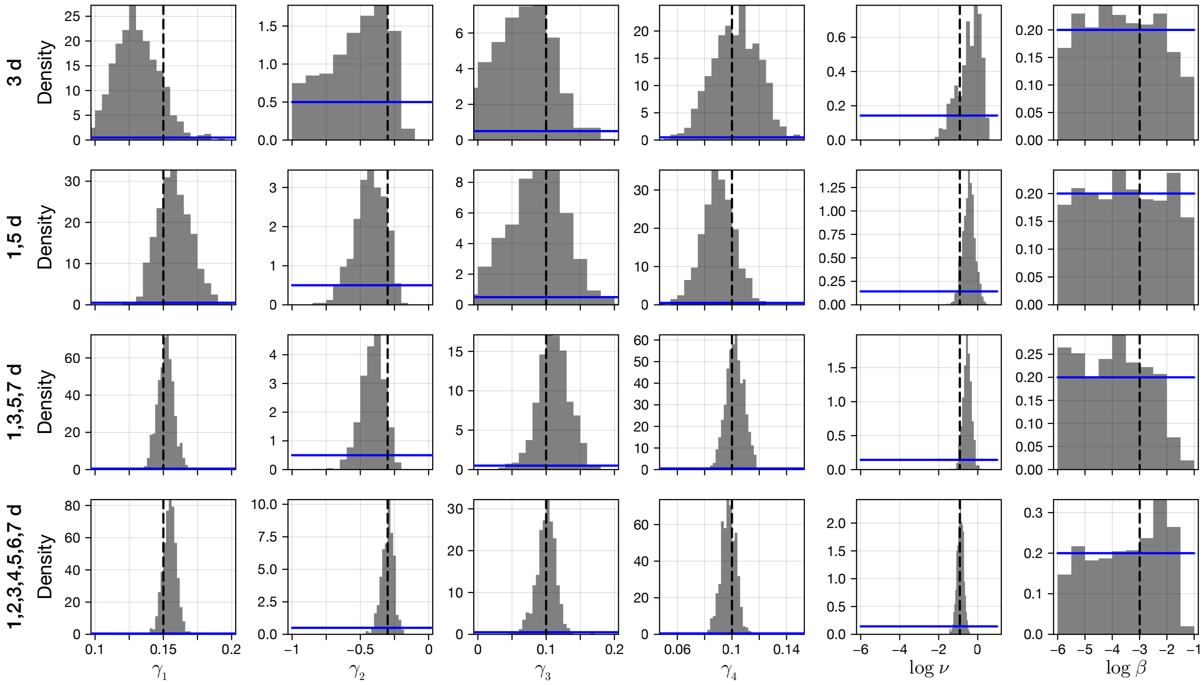

Figure A. Marginal posterior distributions. We show marginal posterior distributions for all parameters, for various sets of observation termination times. Shown also are the marginal prior distributions (blue) and the true values (black dashed).
