# Supplementary material for: Identifiability of phenotypic adaptation from low-cell-count experiments and a stochastic model
Source: PLoS Comput Biol. 2025 Jun 24;21(6):e1013202. doi: 10.1371/journal.pcbi.1013202 (PMC12204626; doi:10.1371/journal.pcbi.1013202)
Supplement: S3 File — (PDF) [file pcbi.1013202.s003.pdf]

S3 CME for discrete model

Let $q(n_1, n_2, t)$ be the density for n_1 sensitive cells and n_2 resistant cells. Let λ_i denote the proliferation rate of each cell population, and δ_i denote the death rate of each cell population (one of which will be zero). We have that

$$\begin{aligned}\frac{\mathrm{d}q(n_1, n_2, t)}{\mathrm{d}t} &= (n_1 - 1)\lambda_1 q(n_1 - 1, n_2, t) + (n_1 + 1)\delta_1 q(n_1 + 1, n_2, t)\\ &\quad + (n_2 - 1)\lambda_2 q(n_1, n_2 - 1, t) + (n_2 + 1)\delta_2 q(n_1, n_2 + 1, t)\\ &\quad + n_1 r_{12} q(n_1 + 1, n_2 - 1, t) + n_2 r_{21} q(n_1 - 1, n_2 + 1, t)\\ &\quad - (n_1\lambda_1 + n_1\delta_1 + n_2\lambda_2 + n_2\delta_2) q(n_1, n_2, t).\end{aligned} \tag{1}$$

To obtain the mass function for the total cell count, $q(n, t)$, we consider that

$$q(n, t) = \sum_{n_1=0}^{\infty} q(n_1, n - n_1, t). \tag{2}$$

In practice, we consider a partial sum truncated at $n_1 = 50$, which we find to be sufficient given the maximum cell counts observed in Fig. 8 of the main text.
