# Supplementary material for: Identifiability of phenotypic adaptation from low-cell-count experiments and a stochastic model
Source: PLoS Comput Biol. 2025 Jun 24;21(6):e1013202. doi: 10.1371/journal.pcbi.1013202 (PMC12204626; doi:10.1371/journal.pcbi.1013202)
Supplement: S4 File — (PDF) [file pcbi.1013202.s004.pdf]

S4 Structural identifiability of heterogeneity

In the main text, we observe that the heterogeneity parameter, β, is one-sided practically identifiable. In this section, we repeat the computational experiment associated with Fig. 3 of the main text to investigate a scenario where a large data set is generated that comprises 768 cell proliferation assays (i.e., eight plates), for each condition, at a set of termination times $t = \{0.5\,\mathrm{d}, 1\,\mathrm{d}, 1.5\,\mathrm{d}, \cdots, 6.5\,\mathrm{d}, 7\,\mathrm{d}\}$. Results in Fig. A show that, in this large data-set regime (5,376 proliferation assays equivalent to a total of 56 plates), the diffusivity parameter is practically identifiable, demonstrating that it is a structurally identifiable parameter.

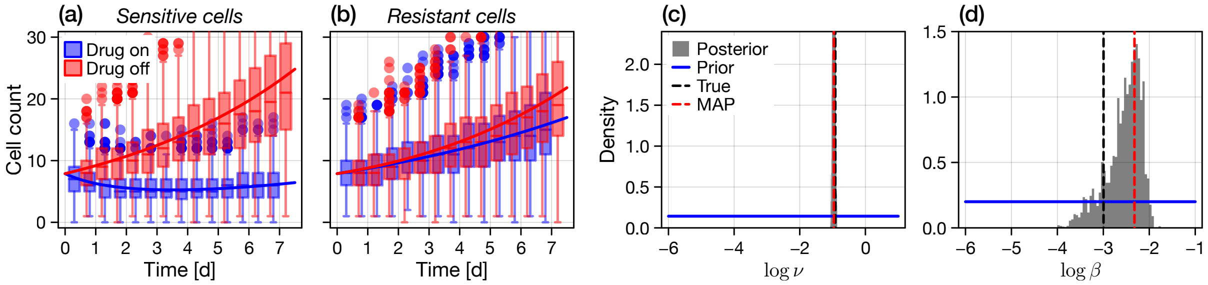

Figure A. Large data set proliferation assay inference. We show marginal posterior distributions for all parameters, for various sets of observation termination times. We perform Bayesian inference on synthetic cell proliferation assay data using the CME as a likelihood. Independent cell count observations ($M = 768$ replicates per condition) are made from experiments conducted with fully sensitive or fully resistant cells, with and without drug, and terminated at $t = \{0.5\,\mathrm{d}, 1\,\mathrm{d}, 1.5\,\mathrm{d}, \cdots, 6.5\,\mathrm{d}, 7\,\mathrm{d}\}$. (a–b) Synthetic proliferation assay cell count data (box plots with outliers shown as discs) and the model predicted mean cell count at the MAP (solid lines). (c–d) Posterior distributions for the logarithm of v, the adaptation speed, and β, the diffusivity. Shown also is the uniform prior (blue), the true value (black dashed), and the MAP (red dashed). In this regime, both the adaptation speed and diffusivity are identifiable.
