# Supplementary material for: Identifiability of phenotypic adaptation from low-cell-count experiments and a stochastic model
Source: PLoS Comput Biol. 2025 Jun 24;21(6):e1013202. doi: 10.1371/journal.pcbi.1013202 (PMC12204626; doi:10.1371/journal.pcbi.1013202)
Supplement: S5 File — (PDF) [file pcbi.1013202.s005.pdf]

S5 Large data set inference with noisy data

In the main text, and in all supplementary results hitherto, we have studied a scenario where exact cell counts are made. We find in S4 File that, in this effectively noise-free regime, the heterogenity parameter is identifiable provided that a sufficiently large data set is available. We now revisit this assumption by assuming that cell count observations, denoted y, are subject to binomial noise such that

$$y \,|\, n \sim \text{Truncated}\left(\text{Binomial}(M(n), 0.5) - \frac{M(n)}{2} + n, 0, \infty\right), \tag{1}$$

where n is the exact cell count, and $M(n)$ is chosen such that the standard deviation of the noise term scales with the cell count (note that for the right hand side of Eq. (1) to be valid, we require that $M(n)$ is always even, such that the noise term $\text{Binomial}(M(n), 0.5) - M(n)/2$ is symmetric about zero. In this section, we set

$$M(n) = 2 \cdot \text{round}\left(\frac{4\alpha^2 n^2 + n_0}{2}\right), \tag{2}$$

such that the noise comprises a count independent term, n_0 (i.e., noise present even in very low cell count observations arising from, e.g., cellular debris), and a count dependent term of magnitude α which scales such that the standard deviation of the noise term is approximately αn for large n. For the results that follow, we set $\alpha = 0.1$ and $n_0 = 5$; for these parameter values we demonstrate the noise distribution and resultant observed cell count distribution in Fig. A. While complex, this choice of discrete noise model accounts for both over and under counting (for example, an automated counting algorithm that both misses cells, and misclassifies cellular debris as cells).

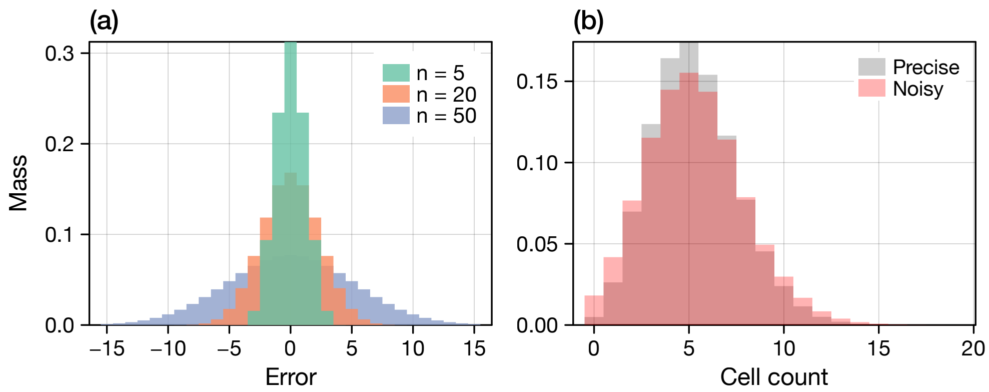

Figure A. Observation noise model. (a) We consider cell count observations subject to additive Binomial error that scales with the cell count, n, according to Eq. (2). (b) Comparison between precise (black) and noisy (red) cell count distributions from the CME.

Following the construction of the statistical observation noise model, we reproduce the large data set results of S4 File in the case that only noisy observations are available. Priors are given in Table A and both fits and marginal posterior distributions in S4 File. Results in Fig. B demonstrate that the diffusivity parameter is again only one-sided identifiable; even from a large data set, we cannot distinguish heterogeneity from observation noise.

Table A. Prior distributions for the noise distribution parameters used in the inference of noisy data.

Parameter	Prior
α	Uniform$(0, 1)$
n_0	Uniform$(0, 10)$

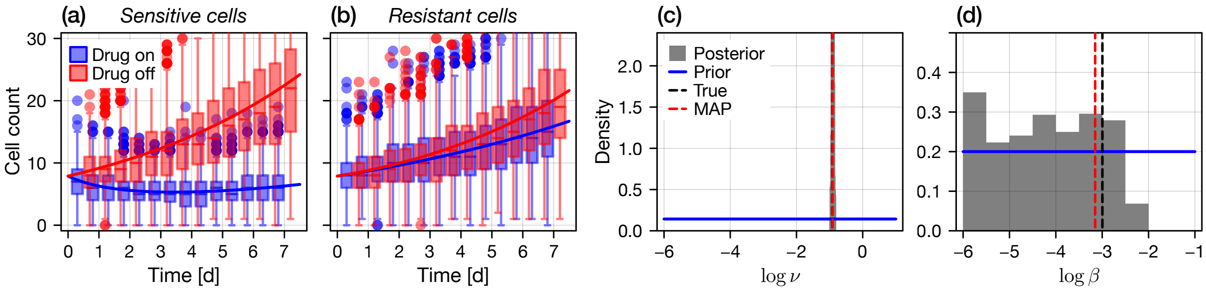

Figure B. Large data set proliferation assay inference with noisy data. We reproduce the results in S4 File in the case that cell count observations are subject to noise of the form given in Eq. (1). The noise parameters α and n_0 are assumed to be unknown, with priors given in Table A.
