# Supplementary material for: Identifiability of phenotypic adaptation from low-cell-count experiments and a stochastic model
Source: PLoS Comput Biol. 2025 Jun 24;21(6):e1013202. doi: 10.1371/journal.pcbi.1013202 (PMC12204626; doi:10.1371/journal.pcbi.1013202)
Supplement: S6 File — (PDF) [file pcbi.1013202.s006.pdf]

S6 Inference with correlated data

For simplicity, in the main document we assume that all cell count observations are independent. For a cell proliferation assay, this is often not true: rather, observations of the *same* cell proliferation assay are made at multiple time points.

In the case that measurements are non-subject to measurement noise, an analogous log-likelihood can be constructed using the chemical master equation. Denote $0 < t_1 < t_2 < \ldots < t_K$ the set of observation times, and $N_1, N_2, \ldots, N_K$ a corresponding set of observed counts. The contribution to the likelihood can be simplified by applying the Markov property of the process, such that

$$\mathbb{P}(N_1, N_2, \ldots, N_K) = \mathbb{P}(N_K|N_{K-1})\mathbb{P}(N_{K-1}|N_{K-2})\cdots\mathbb{P}(N_1). \tag{1}$$

The term $\mathbb{P}(N_1)$ is simply given by a numerical solution to the chemical master equation at $t = t_1$. Other terms $\mathbb{P}(N_k|N_{k-1})$ can similarly be obtained by the solution to a chemical master equation at $t = t_k$ subject to the initial condition $N(t_{k-1}) = N_{k-1}$.

In Fig. A, we reproduce Fig. 3 of the main document in the case that $M = 192$ wells are used per condition (a total of two 96-well plates), all observed at the four time points. Thus, the same number of plates are used per condition, but a set of four correlated observations are made of each well. Results in Fig. A show that β remains one-sided identifiable.

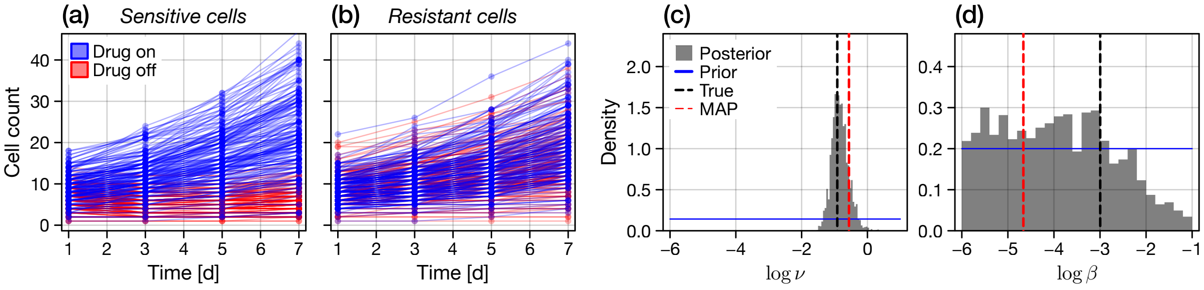

Figure A. Inference with correlated data. We reproduce the results in Fig. 3 of the main document in the case that the data are correlated between observation times.
