# Supplementary material for: Identifiability of phenotypic adaptation from low-cell-count experiments and a stochastic model
Source: PLoS Comput Biol. 2025 Jun 24;21(6):e1013202. doi: 10.1371/journal.pcbi.1013202 (PMC12204626; doi:10.1371/journal.pcbi.1013202)
Supplement: Attachment — Submitted filename: review.pdf [file pcbi.1013202.s007.pdf]

Review of "Identifiability of heterogeneous phenotype adaptation from low-cell-count experiments and a stochastic model"

Summary

Emergence of treatment resistance is a major challenge to cancer therapy. While a plethora of mathematical models for this phenomenon exist, the identification of suitable mathematical models and parameters is an open question. This work examines the identifiability of parameters and models concerning heterogeneous phenotypic adaptation from low-cell-count experiments.

The authors define a stochastic model for phenotypic adaptation in a continuous phenotype space corresponding to fully sensitive to fully resistant phenotypes. Individual cells' phenotypes are governed by a stochastic differential equation which quantifies the resistance/sensitivity of that cell to treatment. Depending on the phenotype a growth rate is assigned in an affine-linear manner depending on the presence of treatment.

After presenting their model and its equivalent formulations using a partial differential equation and a chemical master equation, a log-likelihood function based on the chemical master equation is deduced. Applying a Bayesian parameter inference scheme based on the log-likelihood function, the authors show that it is not possible to identify phenotypic heterogeneity using only cell-count data at different time points.

However, when death and proliferation event times are collected, phenotype heterogeneity becomes identifiable, given that measurement uncertainties are small enough. Motivated by experiments in which a cell proliferation marker was linearly correlated with cell counts, the authors show that this does not give information if and to what extent there is phenotypic heterogeneity.

Due to the identifiability of all other parameters, the authors also investigate the dependency on the functional form of the adaptation velocity. They find that the adaptation velocity has only minor effect on the resulting cell counts.

Lastly, the authors argue that continuous and discrete (binary) phenotype definitions are not distinguishable from the cell-counts only.

Major observations

Overall, the course of arguments is very clear throughout the paper. The hypotheses on the model and the derived mathematical equations are made

clear and precisely defined. However, there are some issues that should be addressed before publication.

- The model definition for the individual-based model should be stated more clearly. Currently it is not clear if the net growth rate λ defined is individual for each cell, depending on each cell's phenotype, or if the net cellular growth rate is only used at the population level. For an individual based model, I would expect phenotype-dependent birth and death rates.
- The methods which were used for inference of the parameters depend on the choice of settings which are not made clear throughout the text. In particular for the Bayesian inference framework there should be a statement on the number of iterations and further settings used in the adaptive algorithm. Also, there should be a comment on how NLopt is used for identifying the maximum a posteriori estimate (MAP).
- In section 3.2 the used log-likelihood function is not explicitly specified. However, this would be needed to understand how the event-timing enables identifiability.
- Lastly, β relates to the standard deviation of the phenotype probability density, and thereby to the variation in net growth rate. However, the cell count is expected to only depend on the mean of the growth rate, which is why the results of unidentifiability of the standard deviation in most cases is not too surprising. To make this more clear, assume we have any distribution (continuous or discrete phenotype space, assuming here continuous x) $u(x,t)$ and a net proliferation rate $r(x)$ with $\partial_t u(x,t) = r(x)u(x) + \text{phenotypic switch terms}$. Then, the total cell number changes as $N(t) = \int p(x,t)dx$, $\frac{d}{dt}N(t) = \langle r\rangle(t)N(t)$, with $\langle r\rangle = \int r(x)p(x,t)/N(t)$ (since phenotypic switch terms do not change $N(t)$). This suggests that it is only possible to infer $r(x)$ and, by extension, the mean phenotype from pure cell count observations. The mean proliferation rate $\langle r\rangle(t)$ follows $\frac{d}{dt}\langle r\rangle(t) = \langle \Delta r^2\rangle(t) + \text{terms related to the phenotypic switch}$ (Fisher's fundamental theorem of natural selection). This suggests that to infer the phenotypic heterogeneity (related to the variance), information about the mean growth rate over time is needed. This likely explains the authors' result in section 3.2, since the event timing data is clearly related to the mean growth rate.
- Overall, I believe the paper's potential impact could be increased if the authors would include a "recipe to infer phenotypic heterogeneity", i.e., a guide that could be used in correspondence with experimental partners, which includes clear requirements about their experiments to be able to

infer phenotypic plasticity, for example number of experiments, observables (cell counts, event timings, mean growth rate?). This could be part of the conclusion/discussion.

Minor observations

- Section 1, second paragraph: The authors state that there are 'seemingly opposing observations that resistance corresponds to a well-defined discrete phenotype and a continuous spectrum of phenotypes'. Why is this only "seemingly opposing"? Either it is discrete or it is continuous. If this is some foreshadowing on the result that either definition can be used during mathematical modeling, please make this clear.
- Second paragraph after figure 1, second sentence: include explicit citation for Hamis et al. ([32]).
- Figure 2:
  - What does 'addicted' mean? Should it be 'resistant'?
  - What does "birth" (circle) and "death" (square) mean here?
  - The colored labels in (e) are barely visible, please improve.
- Section 2.2: "Experiments are initiated with a sample of cells from a zero-net-growth stationary distribution" - why?
- Equations (9) and (10): Which $p(x,t)$ is used here? The one obtained from solving the PDE? Please specify. The equation could be simplified if the integral is restricted to the interval $[0,1]$.
- Section 2.4, first paragraph:
  - Typo 'primilarly' $\to$ 'primarily'.
  - "As we are primarily interested in parameter and model identifiability, we initiated each chain using the "true" set of parameter values that are used to generate synthetic data." Unclear what you mean here, please explain.
- Figure 3: I assume the deviations between the fitted model and the synthetic dataset originates from the (consciously chosen) low amount of input data?
- Figure 4:
  - Use different colors for different β, but the same across both plots.
  - Caption: In the second to last sentence, there is a closing bracket missing.
  - "heterogenity" $\to$ "heterogeneity".
  - You write "In the absence of heterogenity (i.e., for $\beta \to 0$), proliferation and death events cannot occur simultaneously in a population." This

seems a limitation of your model formulation (you discuss this limitation in the discussion section, but it should be mentioned earlier in the main text, somewhere during model definition). This is related to this sentence in section 3.1: "The most obvious consequence of the $\beta = 0$ parameter regime is that proliferation and death cannot occur simultaneously:"

- Section 3.3, first paragraph: typo: 'cyometry' $\rightarrow$ 'cytometry'.
- Section 3.4, first paragraph: $v(x) := v(x, 1)$ (not the other way around). Equation (16): why this form for the wave speed and not any other general formulation?
- Section 4:
    - You discuss the limitation that proliferation is modeled as a Markov process. Maybe relevant to this point are works by Gavagnin and Yates who investigated models with realistic cell cycle times. E.g., Gavagnin et al, "The invasion speed of cell migration models with realistic cell cycle time distributions", J. Th. B. 2019.
    - Last paragraph: Message of the first sentence is that heterogeneous phenotype adaption is identifiable, however in the same paragraph it is stated that heterogeneity is non-identifiable. Is that because $v(x)$ is identifiable and β is not? Please make that more clear. This also relates to my last major observation that the paper's story is not fully clear.
