# Supplementary material for: Identifiability of phenotypic adaptation from low-cell-count experiments and a stochastic model
Source: PLoS Comput Biol. 2025 Jun 24;21(6):e1013202. doi: 10.1371/journal.pcbi.1013202 (PMC12204626; doi:10.1371/journal.pcbi.1013202)
Supplement: Attachment — Submitted filename: responses.pdf [file pcbi.1013202.s008.pdf]

Referee 1

This paper investigates the identifiability of key parameters governing heterogeneous phenotype adaptation in cancer, using a stochastic individual-based model (IBM) applied to low-cell-count proliferation assays. The paper is well written with a clear and well-structured mathematical framework.

The authors take a rigorous approach to the problem of identifiability, systematically analysing different experimental regimes and the conclusions are justified by the mathematical findings. Overall, I believe this paper is a strong contribution to the study of adaptive resistance in cancer, providing valuable insights into the identifiability of key model parameters. However, there are a few minor aspects that in my opinion need attention before publishing the paper.

Response: We thank the referee for their supportive and encouraging report. We now address their individual comments.

R1.1 The main aim of the paper is to "assess identifiability of key model parameters". However, the authors fail to introduce identifiability properly, e.g., to explain why is it important to consider it, or to reference previous literature satisfactorily. In particular I refer to the work by M.P. Saccomani, for example:

- G Bellu, MP Saccomani, S Audoly, L D'Angiò. DAISY: A new software tool to test global identifiability of biological and physiological systems. Computer methods and programs in biomedicine 88 (1), 52-61
- S Audoly, G Bellu, L D'Angio, MP Saccomani, C Cobelli. Global identifiability of nonlinear models of biological systems. IEEE Transactions on biomedical engineering 48 (1), 55-65

Response: We agree that we were not entirely clear regarding our definition of parameter identifiability in the introduction. In the revised introduction, we now clearly state that we are primarily interested in "practical identifiability" (referred to throughout as simply "identifiability" from that point on). It does appear to be the case that the models are (in theory) structurally identifiable, and thus we agree that it is important to draw a clear distinction and motivate our analysis of practical identifiability. References to identifiability have been tweaked throughout, with a clear definition and motivation added to the introduction (Page 2).

R1.2 The study would benefit from a broader discussion of alternative sources of heterogeneity, practical experimental constraints, and potential ways to distinguish discrete and continuous adaptation in real-world data. I would suggest adding this aspects to the Discussion and Conclusion section.

Response: We are now much more clear in the introduction about what sources of heterogeneity we are primarily concerned with (Page 3). We also now reiterate these sources much more clearly within the context of our results in the discussion (Page 20).

In part, our findings provide evidence to suggest that discrete and continuous adaptation are practically indistinguishable from population-level data (i.e., cell counts). Single-cell information is needed to distinguish these strategies. However, our results do demonstrate a realistic experimental

design that could be used to parameterise our model of adaptation. We have revised the discussion to make the practical recommendations that follow from our analysis more clear (Page 20).

R1.3 The authors made the code available. However, the README file is of no help to understand how to run the code. Is there an order in which the files should be run? Is there a main file? What packages are needed? and so on. Please improve the code accessibility.

Response: We have revised the documentation on the GitHub repository to make it clear how each figure can be reproduced. We have also created a Julia environment so that a user can install all required packages with the `instantiate` command.

R1.4 Finally some minor suggested changes are:

(a) At pg 10 the authors write " ... all growth rate parameters are practically identifiable." What does that mean are they or not? I would remove the "practically".

Response: We now define the term 'practical identifiability' in the introduction of the revised manuscript as part of our response to Comment R1.1.

(b) At pg 11 there is a typo, soley should be solely.

Response: We have corrected this typo (Page 12).

(c) I suggest using "Poisson" instead of "Po" when indicating a Poisson distribution.

Response: We have made this change (Page 12).

(d) At pg.14 it is unclear where the expression of $v(x)$ comes from. Explain in more detail the function choice. Also, there is a typo and asgn, should be sgn or sign (if not, explain what you mean by that).

Response: This expression should be read as $a \times \mathrm{sgn}(x)$. We have added a small space between a and $\mathrm{sgn}(x)$ to make this clearer. The functional form of $v(x)$ is chosen to be relatively general (i.e., we can recover standard linear and quadratic velocity functions by setting certain parameters to zero). We have revised the text to make this clear when $v(x)$ is first introduced (Page 15).

Referee 2

Overall, the course of arguments is very clear throughout the paper. The hypotheses on the model and the derived mathematical equations are made clear and precisely defined. However, there are some issues that should be addressed before publication.

Response: We thank referee 2 for their helpful and encouraging report. We now address their specific comments.

R2.1 The model definition for the individual-based model should be stated more clearly. Currently it is not clear if the net growth rate λ defined is individual for each cell, depending on each cell's phenotype, or if the net cellular growth rate is only used at the population level. For an individual based model, I would expect phenotype-dependent birth and death rates.

Response: We agree, this was not immediately clear. We now state clearly that the net cellular growth rate is phenotype-dependent, and only present the net growth rate function as $\lambda(x_i, T_\mathrm{x})$ instead of the (potentially ambiguous) $\lambda(x, T_\mathrm{x})$ (Page 5).

R2.2 The methods which were used for inference of the parameters depend on the choice of settings which are not made clear throughout the text. In particular for the Bayesian inference framework there should be a statement on the number of iterations and further settings used in the adaptive algorithm. Also, there should be a comment on how NLopt is used for identifying the maximum a posteriori estimate (MAP).

Response: We now provide additional details on the algorithms and implementations used. We also state now that we apply the global search algorithm on the posterior density function to find the MAP. Both changes are reflected on Page 10.

R2.3 In section 3.2 the used log-likelihood function is not explicitly specified. However, this would be needed to understand how the event-timing enables identifiability.

Response: We now make it clear that the log-likelihood function is given by the probability mass function of the Poisson distribution in each interval, and that the number of observed events in each interval is statistically independent (Page 12).

R2.4 Lastly, β relates to the standard deviation of the phenotype probability density, and thereby to the variation in net growth rate. However, the cell count is expected to only depend on the mean of the growth rate, which is why the results of unidentifiability of the standard deviation in most cases is not too surprising. To make this more clear, assume we have any distribution (continuous or discrete phenotype space, assuming here continuous x, $u(x,t)$ and a net proliferation rate $r(x)$ with

$$\partial_t u(x,t) = r(x)u(x) + \text{phenotypic switch terms.}$$

Then, the total cell number changes as

$$N(t) = \int u(x,t)\mathrm{d}x, \qquad \frac{\mathrm{d}N(t)}{\mathrm{d}t} = \langle r\rangle(t)N(t),$$

with $\langle r\rangle = \int r(x)u(x,t)/N(t)\mathrm{d}x$ (since phenotypic switch terms do not change $N(t)$). This suggests that it is only possible to infer $r(x)$ and, by extension, the mean phenotype from pure cell count observations. The mean proliferation rate $\langle r\rangle(t)$ follows $\frac{\mathrm{d}\langle r\rangle(t)}{\mathrm{d}t} = \langle\Delta r^2\rangle(t)$ + terms related to the phenotypic switch (fisher's fundamental theorem of natural selection). This suggests that to infer the phenotypic heterogeneity (related to the variance), information about the mean growth rate over time is needed. This likely explains the authors' result in section 3.2, since the event timing data is clearly related to the mean growth rate.

Response: We agree that it is perhaps, in hindsight, obvious that β is practically non-identifiable from reasonable amounts of experimental data. In particular, the average per-capita growth rate only depends on the subpopulation heterogeneity through higher order terms. However, it is routine for both PDE models similar to Eq. (4) to be applied, and for only population-level data to be accessible for model calibration.

R2.5 Overall, I believe the paper's potential impact could be increased if the authors would include a "recipe to infer phenotypic heterogeneity", i.e., a guide that could be used in correspondence with experimental partners, which includes clear requirements about their experiments to be able to infer phenotypic plasticity, for example number of experiments, observables (cell counts, event timings, mean growth rate?). This could be part of the conclusion/discussion.

Response: One of our main conclusions is that it is likely not possible to identify continuous phenotypic heterogeneity from population-level data. However, our results do demonstrate an experimental design sufficient to identify all other key model parameters; we now discuss this point in the revised discussion (Page 20).

R2.6 finally some minor suggested changes are:

(a) Section 1, second paragraph: The authors state that there are "seemingly opposing observations that resistance corresponds to a well-defined discrete phenotype and a continuous spectrum of phenotypes". Why is this only "seemingly opposing"? Either it is discrete or it is continuous. If this is some foreshadowing on the result that either definition can be used during mathematical modeling, please make this clear.

Response: Indeed, we had intended this phrase to foreshadow the result that both models are indistinguishable at the population level. We have removed this, and just state that the observations are "contrasting" (Page 2).

(b) Second paragraph after figure 1, second sentence: include explicit citation for Hamis et al. ([32]).

Response: We now include an explicit citation at every mention of this reference (Page 3).

(c) figure 2:

- What does 'addicted' mean? Should it be 'resistant'?
- What does "birth" (circle) and "death" (square) mean here?
- The colored labels in (e) are barely visible, please improve.

Response: We have corrected all mentions of the term "addicted" to "resistant", and have adjusted the label colours in (e). The legend in (a) indicates that circles correspond to birth events, and squares correspond to death events. We now make it clear in the caption of figure 2 that these are shown in (a) and (b) (Page 7).

(d) Section 2.2: "Experiments are initiated with a sample of cells from a zero- net-growth stationary distribution" - why?

Response: The true initial condition is given as the solution of a boundary value problem (i.e., stationary PDE), to allow for the growth rate to vary with x. This adds computational complexity, and we find makes negligible difference to the overall results.

(e) Equations (9) and (10): Which $p(x,t)$ is used here? The one obtained from solving the PDE? Please specify. The equation could be simplified if the integral is restricted to the interval [0, 1].

Response: We now make it clear in the text following Eq. (10) that $p(x,t)$ is governed by the PDE (Section 2.3). We agree that the integral may be simpler to compute numerically if we worked with an individual based model with a finite phenotype domain. However, we wish to avoid the assumption that the phenotype space is explicitly bounded.

(f) Section 2.4, first paragraph:

i. Typo 'primilarly' $\rightarrow$ 'primarily'.

ii. "As we are primarily interested in parameter and model identifiability, we initiated each chain using the "true" set of parameter values that are used to generate synthetic data." Unclear what you mean here, please explain.

Response:

i. We have thoroughly proofread the manuscript and corrected all typos.

ii. We now expand on our justification for initiating each chain at the "true" parameter values (Page 10).

(g) Figure 3: I assume the deviations between the fitted model and the synthetic dataset originates from the (consciously chosen) low amount of input data?

Response: The fitted model and the synthetic data set do actually agree quite well, although this may not be obvious from figure 3 as it currently stands. We have modified figure 3a and b to show the predicted quantiles, which demonstrate agreement between the fitted model and synthetic data set.

(h) Figure 4:

i. Use different colors for different β, but the same across both plots.

ii. Caption: In the second to last sentence, there is a closing bracket missing.

iii. "heterogenity" $\rightarrow$ "heterogeneity".

iv. You write "In the absence of heterogenity (i.e., for $\beta \rightarrow 0$), proliferation and death events cannot occur simultaneously in a population." This seems a limitation of your model formulation (you discuss this limitation in the discussion section, but it should be mentioned earlier in the main text, somewhere during model definition). This is related to this sentence in section 3.1: "The most obvious consequence of the $\beta = 0$ parameter regime is that proliferation and death cannot occur simultaneously:"

Response:

i. It was our intention to draw a clear distinction between the growth and death rates, however it was confusing having the growth rate change from predominantly green to blue between Figs. 4 and 5. We have adjusted the colour scheme in Fig. 4a so that the growth rate is always shown in a shade of blue (Page 13).

ii., iii. We have corrected these typos and thoroughly proofread the manuscript.

iv. This choice is now explicitly mentioned when the model is first presented (Page 5).

(i) Section 3.3, first paragraph: typo: 'cyometry' $\rightarrow$ 'cytometry'.

Response: We have thoroughly proofread the manuscript and corrected all typos.

(j) Section 3.4, first paragraph: $v(x) := v(x, 1)$ (not the other way around). Equation (16): why this form for the wave speed and not any other general formulation?

Response: To avoid confusion, we now refer to the speed as $u(x)$ where $v(x, 1) = u(x)$ (without a definition). Regarding the formulation, our choice allows a variety of forms, of increasing complexity, by setting various parameter combinations to zero. While higher-order terms could be included, this is likely not necessary (the system behaviour is only dependent on $u(x)$ near the interval $x \in (0, 1)$). We have amended the text to make this all clear (Page 15).

(k) Section 4:

i. You discuss the limitation that proliferation is modeled as a Markov process. Maybe relevant to this point are works by Gavagnin and Yates who investigated models with realistic cell cycle times. E.g., Gavagnin et al, "The invasion speed of cell migration models with realistic cell cycle time distributions", J. Th. B. 2019.

ii. Last paragraph: Message of the first sentence is that heterogeneous phenotype adaption is identifiable, however in the same paragraph it is stated that heterogeneity is non-identifiable. Is that because $v(x)$ is identifiable and β is not? Please make that more clear. This also relates to my last major observation that the paper's story is not fully clear.

Response:

i. We now explicitly mention alternative time-to-proliferation distributions that are applicable for models without adaptation, and cite the suggested reference (Page 20).

ii. We are now much more clear throughout the manuscript, and particular here in the discussion, about what we mean by "heterogeneity". This conclusion is now, in our view, much more clear.

Referee 3

In this study, Browning et al present a computational framework to infer how a population of cancer cells adapts to treatment from time-lapse data. Specifically, the framework examines the role of drug-induced, phenotypic switching of cells, as has recently been observed in several cancer settings. The authors perform simulation experiments to explore to what degree it is possible to infer the growth rates of cells in different phenotypic states, the rates of random and drug-induced switching, and whether or not changes in cell phenotype are continuous or discrete. In doing so, the authors address an important question regarding the calibration of mathematical models of resistance and the design of in vitro experiments that is timely given the growing evidence for the role non-genetic processes in cancer drug resistance evolution.

This is a strong and well written manuscript which will be of great interest to readers of this journal. It is essentially ready for publication, except for three points which I would ask the authors to address to clarify some of the language in the paper. I also have few additional minor suggestions/typos that can be found at the end of my response. Thank you for inviting me to contribute to this manuscript as a reviewer and congratulations to the authors on a great piece of work!

Response: We thank the reviewer for their positive report, and many helpful suggestions. We now address their individual comments.

R3.1 "Adaptive Resistance". I have not heard the term "adaptive resistance" before. I'm happy to accept my ignorance here, but I find this particular term a bit confusing because of the strong history and meaning that the term "adaptation" has in classical evolutionary theory. Specifically, what worries me about "adaptive resistance" is that it confounds whether the adaptation is happening on the individual level (as in scenario you are studying) or on the population level (as assumed in classical evolutionary theory, which can be brought about by genetic changes (Darwinian Model) and/or phenotypic changes (Lamarckian Model)). There isn't great consensus yet around terminology for the drug-induced and heritable phenotypic changes we are seeing in cancer, but in order to better connect with the existing literature I would suggest you to mention some of the terms others have used (specifically the papers you are citing). e.g. "phenotypic switching", "epigenetic plasticity", "drug-induced resistance", or "non-genetic resistance". And if you don't feel strongly, maybe even consider adopting one of those terms instead of introducing a new one.

Response: We agree, there are a multitude of terms in the literature that all refer to short-term, reversible, phenotypic change. In response to this comment, we have changed the phrase "adaptive resistance" to "phenotypic plasticity", which carries a more broadly recognisable meaning. In the introduction, we are also now much clearer on the mechanisms in our model behind the short-term and reversible phenotypic changes (e.g. Page 3).

R3.2 Similarly, I would ask you to change the way in which you use the term "heterogeneity". From what I can tell you use it to describe two related, but distinct phenomena: i) Whether cells exist in 2 discrete states (sensitive and resistant) or in a continuum of states along a sensitive-resistant axis, and ii) whether switching of cells between states with different drug sensitives is random (diffusive) or directed (advective/drug-induced). Firstly, I would argue that since all your models include sensitive and resistant cells in some form they model a "heterogeneous" cell population (at least in the way most cancer biologist would think about it). So, I'm not sure that "heterogenity" is a good word to use for either phenomenon. Regarding Point i), evolutionary biologists use the terms "gradualism" (many small changes) and "saltationism" (few, big changes; I've also heard "step-wise evolution") to distinguish between these scenarios. Perhaps one of those would be clearer? Regarding Point ii) I think this distinction would be worth explicitly highlighting. The question whether phenotypic adaptation happens randomly (drift) or is drug-induced (advection) is one of the key questions in cancer biology at the moment. I think a discussion of this point and how your framework can help to answer this question would be a great addition to your manuscript. See my comment about Page 10 for more details.

Response: We agree, our use of the term "heterogeneity" is confusing, particularly as it is used interchangeably to refer to both the model of phenotype adaption itself (a discrete vs a continuous space) and to the cell-to-cell heterogeneity induced by random (diffusive) changes in phenotype. In response, we have:

(a) Removed "heterogeneity" from the title (Page 1).

(b) Removed all ambiguous uses of the word "heterogeneity" throughout the manuscript

(c) Made the distinction between the two main questions of interest clear (that is, can we distinguish between a discretely structured and a continuously structured phenotype space;

and, can we identify the relative contributions of random and drug-included phenotype changes), particularly when discussing the results.

Changes (ii) and (iii) are reflected throughout the manuscript, in particular in the introduction and discussion. Hence, we do not provide a specific page number.

R3.3 Choice of experimental setup: You conclude that models that assume discrete or continuous phenotypic transitions can not be distinguished, and we're justified in using the simpler 2-state ODE models. That's great news, but I worry that to some extent that this might be confounded to the experimental setup you are studying here. Specifically, the way I understand it all your experiments happen in a constant drug environment (either all on or all off). You present some intermittent data initially, but then partition it into either the on or the off phase. That means you are comparing these models in how they describe the switching from one end of the sensitive-resistant axis to the other (either in a gradual or discrete fashion). We are essentially seeing these cells "roll down a hill" in phenotype space and I think it makes sense that there is little difference in this scenario whether this process is happening in a continuous or step-wise fashion. However, I suspect this might be different under more complex treatment schedules, where it can matter that we have cells half-way between the sensitive and resistant state when we switch the drug concentration. I have added a few papers that demonstrate this. I would be interested in hearing your thoughts about this question. Unless I have misunderstood the implications of your work, I would suggest to add a discussion of this point in the discussion. If you have time I would also be curious to see if your conclusions in Section 3.5 might change if we subjected these cells to intermittent schedules?

- Gunnarsson, E. B., Magnússon, B. V. & Foo, J. Optimal dosing of anti-cancer treatment under drug-induced plasticity. arXiv (2024) doi:10.48550/arxiv.2412.16391.
- Corigliano, M., Bernardo, A. D., Lagomarsino, M. C. & Pompei, S. Optimal treatment for drug-induced cancer persisters involves release periods and intermediate drug doses. bioRxiv 2024.11.29.626082 (2024) doi:10.1101/2024.11.29.626082.
- Smalley, I. et al. Leveraging transcriptional dynamics to improve BRAF inhibitor responses in melanoma. EBioMedicine 48, 178–190 (2019).
- Raatz, M. & Traulsen, A. Promoting extinction or minimizing growth? The impact of treatment on trait trajectories in evolving populations. Evolution 77, 1408–1421 (2023).

Response: This is an excellent point. Our results in Section 3.5 are actually much more general than what was clear in the first iteration of the manuscript. We choose to focus largely on adaptation in a single direction as it is likely to be the case that the adaptation velocity varies between the sensitisation and resistance directions, and thus experiments should in general be conducted in both directions to fully parameterise the system. Under the assumption that the adaptation velocity does in fact vary across adaptation between different pairs of drug concentrations, the equivalence between the discrete and continuous models does hold for any treatment regime. We have modified the text throughout Section 3.5 to make this clear, and have added a set of results to Fig. 8 that show the equivalence for a variety of different intermittent treatment regimes (Page 18).

R3.4 Minor comments:

(a) Fig 1c: could you provide a bit more explanation of what the schematic is showing?

Response: We have added text to the figure caption, and modified the panel itself, to make this clear (Page 4).

(b) Page 3: "intermediate period between resensitisation and addiction" $\rightarrow$ are you referring to "drug addiction" here? If so, I'd suggest explaining what this means and how it relates to this study. I'm also not sure I see evidence for addiction (higher growth rate under drug vs no drug) in your data. Might be simpler and clearer to just say something like "during the re-sensitisation period between days 7-14, when drug was taken off"?

Response: We now refer to drug "resistance", rather than addiction, to be more consistent with our terminology. Additionally, we have taken on board the referees comment and rewritten this sentence (Page 3).

(c) Page 4: "untreated and treated states"; maybe use a different word than "states" to clarify that this to the state of the environment, and NOT the cells.

Response: We thank the referee for this suggestion, and have made the suggested change (Page 5).

(d) Page 5:

i. What is x?

ii. What are γ_i for $i = 1, ..., 4$?

iii. Could you provide a 1 sentence motivation/explanation for the functional form for lambda in Eq 2? Perhaps, you could add a small panel to fig 1 illustrating the assumed relationship between x and lambda.

iv. "Provided that the growth rate is monotonic in x, the functional form of λ is arbitrary since we could, in theory, rescale the phenotypic space in Eq. (1) and thus equivalently the functional form of v". I'm not an expert in this but this feels like a very strong statement. What if lambda is non-linear? Presumably you'd need more than a linear scaling to adjust v to that?

Response:

i. In Eq. 2, and throughout the following paragraph, we have changed the generic variable x to the cell-specific variable x_i to make it clear that we are referring to the cell-specific phenotype index.

ii. We now state that $\lambda(x_i, T_\mathrm{x})$ is parameterised as Eq. 2 (Page 5), to make it clear that γ_i are generic parameters to be estimated.

iii. (and (iv)) To see why this is (in some-sense) arbitrary, consider the passage through growth-rate-space in a deterministic model

$$\frac{\mathrm{d}\lambda}{\mathrm{d}t} = \frac{\mathrm{d}x_i}{\mathrm{d}t}\frac{\mathrm{d}\lambda}{\mathrm{d}x_i} = v(x_i, T_\mathrm{x})\frac{\mathrm{d}\lambda}{\mathrm{d}x_i}. \quad (1)$$

Thus, results depend only on the product of $v(x_i, T_\mathrm{x})$ and $\frac{\mathrm{d}\lambda}{\mathrm{d}x_i}$. Other (monotonic) forms of $\lambda(x_i, T_\mathrm{x})$ can be recovered through different choice of v. While this is not strictly true for $\beta > 0$, given that identifiability is already an issue for the linear choice of v and λ, we expect results to be relatively insensitive to higher-order parameterisations of λ.

(e) Figure 2:

- a) “addicted” $\rightarrow$ should this say “resistant”?
- e & f) It took me a moment to understand what the difference between the two panels is since both include intermittent treatment. could you add a label saying (drug on) and (drug off) to the titles, since this is really what discriminates the two panels?

Response: We have made the suggested changes (Page 7).

(f) Page 8: “The advantage of this approach, compared to a more standard approach that considers an average cell count subject to additive Gaussian noise, is that we account directly for the stochasticity intrinsic to the proliferation death process.” $\rightarrow$ Nice! I think that is a really interesting and useful point.

Response: We thank the referee for their encouraging comment.

(g) Page 9:

i. “kth cell count” $\rightarrow$ do you mean count of cells with phenotype k?

ii. “assuming that measurements are taken at the termination of an experiment and not as a time-series” $\rightarrow$ I got a bit confused when I got to this point. With the time-series images and data you show in fig 1 I was expecting you to use the time-series data. But if I’m understanding this correctly, you are only using a single data point? If that is correct, then I would suggest that you make this assumption clearer in your introduction and discussion too. I don’t think it’s necessarily a limitation, since it’s impressive and useful to know what we can do with a single data point (also thinking about potential in vivo translation/working with more complex experimental setups). But I think it would be important to highlight this more clearly. I also wonder if you were to use the time series data you could address some of the limitations that you identify, since beta will also manifest itself in the time correlation structure?

Response:

i. The cell count is only the number of cells observed in the field-of-view, and the summation over k refers to the fact that multiple independent experiments, with the same experimental parameters, are conducted. We have adjusted the wording in the manuscript to make this clear (Page 10).

ii. While it does add complexity, our method can actually account for temporal correlations using the CME. However, it is not a straightforward extension to non-exact measurements (i.e., the supplementary results that show that we lose identifiability again if cell counts are not exact). We now included supplementary results that show the same issues with identifiability in the case that the correlation structure is accounted for (Fig. S6, and Page 11 of the main document).

(h) Page 10:

i. "one-sided identifiable": I like this term and your thoughtful analysis here. With that said, I wonder if you're being overly pessimistic? Yes, you can't set a lower bound, but isn't the upper bound more interesting anyhow in answering the question of how much random drift contributes to the observed dynamics? By being able to say it's less than x and comparing that bound to nu we can deduce how much each of the processes are driving the adaptation.

ii. "Thus, we expect, from cell count data alone, models with phenotypic heterogenity to be indistinguishable from models with deterministic adaptation (the $\beta = 0$ scenario)" $\rightarrow$ From what I understand what you mean to say here is that you can not distinguish between a scenario in which adaptation is driven purely by drug-driven phenotype switching, and a scenario in which there is both drug-driven switching (the advection term) and random switching (diffusion term; cells can move both up and down the phenotype axis, at a rate that's independent of drug). If this interpretation is correct, then I wonder if this really is a helpful question to ask? Wouldn't one always expect there to be a certain degree of "noise" in biological processes? When, in practice, would we expect a process to be full deterministic? If I may, I would argue that would be more helpful to know is the relative balance between noise and drug-induced switching. Specifically, in the setting of drug resistance evolution, the key question would be: is adaptation happening through random switching, or is it drug-induced, i.e. how strong is the contribution of noise/drift relative to the movement caused by the drug-induced switching/advection term? Could you comment on this, and clarify in the text here what exactly you mean with your statement and what it's practical implications are? Related to my major point, may I also suggest to use a different word than "heterogeneity". I'd argue that you have heterogeneity (sensitive and resistant cell types) even if beta=0. If you have time, it might also be interesting to explore how well your approach can distinguish the two evolutionary scenarios I've outlined above (drug-induced (advective) vs random (diffusive) switching). This would be of great practical interest and would strengthen the potential impact of your work.

Response:

i. We use the standard term "one-sided identifiable" to be clear that we can only establish an upper bound for β. Essentially, we do establish that β cannot be too large, but that (more importantly) it could potentially be zero. That is, we cannot distinguish the $\beta = 0$ regime from a $\beta > 0$ regime.

ii. In response to Comment R3.2, we are now much clearer on what we mean by "heterogeneity". As a consequence, we now more clearly discuss how the non-identifiability of β relates to establishing the relatively contribution of random diffusive switching to drug-induced switching.

(i) Figure 3:

i. "model predicted mean cell count" $\rightarrow$ which model is this referring to? Could yo reference the equation here for clarity?

ii. c & d? From what I understand your inference method only uses the data from a single time point ("end point") for inference. So while all the data in a and b are available in principle, your estimates in c and d only use one of the time points. Which time point is it? And, could you also comment on what effect the choice of time point has on our ability to infer parameters? Is there a optimal length of time one should conduct this experiment over?

Response:

i. There is only one model in the paper. We have modified the text to make it clear that the median cell count is computed using the chemical master equation (Page 12). Note that now the median is used in response to Comment R2.6g.

ii. All inference results use data from all time points, it is just that all data are independent (for more on this, see our response to Comment R3.4g). To make this clear, we have added text following Eq. (13) to reiterate that the summations are over all conditions, time points, and experimental replicates.

(j) Figure 4: missing closing bracket after "maximum proliferation rate"

Response: We have fixed this typo (Page 13).

(k) Page 11:

i. "most obvious consequence of the $\beta = 0$ parameter regime is that proliferation and death cannot occur simultaneously" $\to$ why is that? I'm not sure I'm following. Sorry if I'm missing something. Could you provide more detail here?

ii. "proliferation-death transition" $\to$ Where is that happening? Could you indicate that in fig 4?

iii. "soley" $\to$ typo

iv. "Under the current model formulation, in which heterogeneity is driven soley by diffusion through the phenotypic space, it is only in the regime where $\beta > 0$ that we will ever see proliferation and death events occur simultaneously." $\to$ Related to my major points, I think "heterogeneity" is a confusing word to use here, since you have the possibility of different cell states even without beta. And related to my question above, it is not clear to me why we can't have proliferation and death events at the same time.

Response:

i. In our model, a positive net growth rate corresponds solely to proliferation. Since a single phenotype has a single net growth rate, the sign of this net growth rate will determine whether the cell is able to proliferate or die (but not both). We have added text at the relevant part of Section 3.1 to make this clear (Page 11).

ii. Our response follows (i). The transition occurs when $r_{\text{prol}}(t) = 0$. As this is only relevant for the $\beta = 0$ case where all cells have identical phenotype, it is our view that it may cause confusion if this transition is indicated for $\beta = 0$, which is not considered in the figure.

iii. We have corrected this typo, and thoroughly proofread the manuscript.

iv. We agree that heterogeneity in our model is only driven by phenotype; all other cellular characteristics are, in our model, fixed (homogeneous). The question of other sources

of heterogeneity is an active, parallel area of research for us; indeed, our findings that continuous phenotypic transitions are indistinguishable from discrete transitions may aid model development in this area by shifting the focus back to ODE models. However, it is precisely PDE models like Eq. (3) that are referred to as accounting for heterogeneity in the mathematical literature. Therefore, we choose to keep the term "heterogeneity", albeit with a much clearer qualification, in the introduction and throughout the paper. See also our response to Comment R3.2 for further details.

(l) Page 12:

i. $\delta t = 0.035 \to$ Can you add units? This will be useful to understand what your bin size means practically and how it compares to standard imaging frequencies.

ii. "previousl in Fig 5a" $\to$ did you mean to reference a different figure here?

iii. "Clearly, heterogeneity is now identifiable; estimates of β can be drawn precisely." $\to$ see major comment around your use of the word "heterogeneity".

Response:

i. We have added units to make it clear that the bin width is small relative to all other timescales (Page 12).

ii. This original wording was confusing. We are simply referring to the transition between proliferation and death that can be seen in Fig. 5a. We have adjusted the text accordingly (Page 13).

iii. We refer to our responses to Comments R3.2 and R3.4k.

(m) Page 13:

i. $M_i \to$ What's the subscript i referring to?

ii. Figure 6: It looks like the value you've assumed for sigma is larger than that of beta. It's hard to compare these two values directly, but could it be that the level of noise you're assuming here in the markers is higher than the noise that is generated by the intrinsic stochasticity in the cell dynamics? If so, could that be the reason why we cannot identify beta? What happens if sigma = 0?

iii. Isn't beta one-sided identifiable again?

Response:

i. We now make it clear that, as with the subscript in the term x_i, we refer to the ith cell (Page 14).

ii. We note that the units of β and σ are always not directly comparable. As $\sigma \to 0$, the correlation between the growth rate and the marker measurement will become perfect; that is, the growth rate would be observed directly, as in Fig. 5. If it was the case that $\sigma = 0$ (or sufficiently small) β would be identifiable. However, we do not expect this to be the case with any experimental marker of proliferation. We now state this in the revised manuscript (Page 14).

iii. Yes, β is one-sided identifiable (and, therefore, non-identifiable). To avoid confusion with this terminology, we are now clearer on this point (Page 14).

(n) Page 14:

i. Equation 16: what's "asign(x)"? Could you define that function? It looks like it might be a typo and is meant to read $a * \mathrm{sign}(x)$; even so I think it would be helpful to define what sign(x) is.

ii. $a = c = d = 0$: why are you using this as a test case? Is there particular practical or theoretical motivation? It seems odd to introduce a model with 4 parameters, but then discard 3 of them in the application.

Response:

i. We have added a space between a and the function $\mathrm{sgn}(\cdot)$ to make it clear that we are referring to $a \times \mathrm{sgn}(\cdot)$. Furthermore, we now state clearly that $\mathrm{sgn}(\cdot)$ is the sign function (Page 15).

ii. We use this as a test case as it is the model considered throughout the rest of the manuscript; that is, the well-known Ornstein-Uhlenbeck model. We now make this clear through a reference back to the equation for $v(x_i, T_\mathrm{x})$, to which we now associate with a number (Page 16).

(o) Figure 7: what is $x(t)$? Is that the average phenotype?

Response: As the population is homogeneous, $x(t)$ is both the only phenotype and the average phenotype. We now state this at the start of Section 3.4 (Page 14).

(p) Page 15:

i. "we compare the likelihood at the MLE (equivalently, the MAP) for a = 0, denoted by theta[a]" $\rightarrow$ this makes it sound like you're denoting the likelihood by theta[a], when from what I understand you theta is meant to denote the parameter set?

ii. "we compare the identified adaptation velocities ... to the full model" $\rightarrow$ what's the "full" model? Is that the ground truth model?

Response:

i. Yes, we are referring to the MLE as $\boldsymbol{\theta}_{[a]}$. Given that this is confusing, and that the notation is never used again, we have removed it from the revised manuscript (Page 16).

ii. The full model just has no non-zero parameters. We have adjusted the text to make this clear (Page 16).

(q) Section 3.5. Related to my major comment: how much do the conclusions drawn in this section depend on your assumption that lambda is linearly correlated with x, and that we only have data from experiments under constant treatment conditions (e.g. all on or all off)?

Response: We refer back to our full response to Comment R3.3. In summary, these results do not depend at all on whether λ is linearly correlated with x (we derive a general $\lambda(\cdot)$ which is equivalent to the binary model). We have also chosen the treatment regime arbitrarily: the findings hold whether the drug is on or off for any period of time (and, therefore, by extension any combination; e.g. intermittent therapy).

(r) Page 17:

i. “may arise as a practical limitation that prevents heterogeneity from becoming practically identifiable” → related to my major comments and my comment on page 10: isn’t it useful to be able to put an upper bound on how much noise is contributing? If it’s fairly small that would give an indication that drug-induced adaptation plays a significant role in this process.

ii. “we cannot distinguish be- tween a heterogeneous and a homogeneous model” → related to my major comment: I’d strongly argue to use different terminology. Both models include heterogeneity in that there are cells in different cell state. It’s just the number of these states that is different (gradualism vs saltatism).

iii. “sources of model misspecification” → by whom? It’s unclear if this is referring to the field as a whole or to your work.

iv. “large-cell-count experiments” → large in what way? Long time? Large seeding numbers? Many replicates?

v. “Given that adaptation occurs on a similar timescale as proliferation, what an appropriate time-to-proliferation distribution would be is entirely unclear.” → Love it! If you are interested in pursuing this you might enjoy the CFSE tagging literature. See e.g. these two papers as intros:

- Miao, H., Jin, X., Perelson, A. S. & Wu, H. Evaluation of Multitype Mathematical Models for CFSE-Labeling Experiment Data. Bulletin of Mathematical Biology 74, 300–326 (2012).
- Boer, R. J. D. & Perelson, A. S. Estimating division and death rates from CFSE data. Journal of Computational and Applied Mathematics 184, 140–164 (2005).

Response:

i. By this, we are not referring to noise but to whether individual cells are observed to proliferate sufficiently many times during the adaptation phase. We have adjusted the text accordingly (Page 19).

ii. We respectfully disagree with this comment, although admit we should include the qualifier that it is the homogeneous model of continuous adaptation we refer to. In our model, if $\beta = 0$, all cells are identical: a homogeneous population. We have adjusted the text to make it clear we only refer to the model of continuous adaptation (Page 19).

iii. While we do think that these sources of model misspecification likely apply very broadly throughout the literature, we wish to focus on our model. We have adjusted the text to state this (Page 19).

iv. We have adjusted the text: we refer to experiments initiated with a large number of cells, for which exponential growth may no longer be appropriate (Page 19).

v. We thank the referee for drawing these references to our attention. Indeed, as proliferation is the result of a large chain of cellular events, what a changing “proliferation rate” means very likely involves significant (future) investigation.

(s) Page 18:

i. “where individual cells have a unique growth rate when both fully sensitive and fully adapted” → wait, I though each x corresponds to a unique lambda so that each state has a unique rate associated with it?

ii. " significant to the mathematical modelling community, and implies that population-level behaviours (including, importantly, the response of systems to drugs and the design of adaptive therapies) are well characterised by homogeneous ODE models" $\rightarrow$ I find this a very helpful way of putting your results into context. And I find it a comforting result to read. With that said, as mentioned in my major comments, I worry that this equivalence might start to break down in the case of intermittent schedules where the cells don't get time to full transition into the other state before the dose is changed again. In this case it feels like the two types of approaches might give different results.

Response:

i. We have adjusted the wording to make this clear. We refer here essentially to what the referee refers to in Comment R3.2: variation between cells with an otherwise identical "phenotype". That is, variation in the proliferation rates across cells where $x_i = 1$. We have reworded this sentence to make this clear (Page 20).

ii. While our findings can only be taken within the context our our modelling assumptions, we do actually expect our results to hold for intermittent treatment schedules where the cells don't get to fully transition. This may not be true if other types of heterogeneity are present; however, given the difficulty of distinguishing between continuous and discrete models of adaptation within our simple framework, we expect it to be practically difficult to establish continuous heterogeneity in more complicated models.

(t) Supplementary material Figure S2: how are you combining data from different time points when you are doing the inference?

Response: Please see our response to comment R3.4g(ii).

(u) Figure S3 (and S5): Can you add a label to indicate that a) and b) are sensitive and resistant cells respectively?

Response: Great suggestion, we have made this change in the supplementary material.

(v) Figure S4: "re reproduce the results" $\rightarrow$ "we repeat the (computational) experiment"? I got confused by "reproduce" and thought you were showing the same data again.

Response: We agree, as the experiment is run "again", it is clearer to use the word "repeat" (Page 5).

(w) Section S5:

- Could you provide some more justification/motivation for why you chose this error models and that particular set of parameters? What is the underlying biological motivation?
- Isn't beta one-sided identifiable again?

Response: A simpler choice of noise model may be to assume that each cell has a probability p of being counted, such that the observed number of cells is given by $y|n \sim \text{Binomial}(n, p)$. However, this model only allows for undercounting (i.e., an automated algorithm that misses cells) and not overcounting (i.e., misclassification of debris as cells). We have added text to

the supplementary material to make this clear (Page 6). Finally, we agree that it is clearer to state that β is only one-sided identifiable here, and have adjusted the supplementary text accordingly (Page 7).
